# Evaluation of the effectiveness of foot-and-mouth disease vaccination of animals in the buffer zone of the Republic of Armenia in 2016–2020

**DOI:** 10.1186/s12917-023-03728-8

**Published:** 2023-09-29

**Authors:** Satenik Kharatyan, Khachik Sargsyan, Hasmik Elbakyan, Tigran Markosyan, Pertsh Tumanyan, Varduhi Hakobyan, Vazgen Sargsyan, Manvel Badalyan, Gayane Chobanyan, Jenna E. Achenbach

**Affiliations:** 1 Scientific Center for Risks Assessment and Analysis in Food Safety Area of the Ministry of Economy of the Republic of Armenia (RA), 107/2 Masis Highway, Shengavit, Yerevan, 0071 Republic of Armenia; 2 Reference Laboratory for Especially Dangerous Pathogens of the Republican Veterinary and Phytosanitary Laboratory Services Center of the Food Safety Inspection Body of the RA, 12 Erebuni Street, 0041 Yerevan, Republic of Armenia; 3 Armenian National Agrarian University, 74 Teryan Street, 0009 Yerevan, Republic of Armenia; 4 Battelle Memorial Institute, 1001 Research Park Boulevard, Town Center Two, Suite 400, 22911 Charlottesville, VA USA

**Keywords:** Control measures, Foot-and-mouth disease, Immunity, Prevention, Ruminants, Vaccination

## Abstract

**Background:**

Foot-and-mouth disease (FMD) is a high impact viral disease of livestock for which vaccines are extensively used for limiting the spread of infection. Armenia shares a border with both Turkey and Iran where FMD is endemic, making vaccination an important component of Armenia’s control strategy. Additionally, Armenian veterinary services utilize both passive and active monitoring for prevention control.

**Methods:**

We sought to determine the immune status of animals vaccinated against FMD and to evaluate the effectiveness of our vaccination policy in Armenia. This was conducted in three regions including Shirak, Armavir, and Ararat Region which are located in the buffer zones that border Turkey and Iran. Through active monitoring in 2020, we studied blood serum samples from cattle and sheep using an enzyme immunoassay to determine the level of immune animals in these regions following the use of a polyvalent inactivated vaccine containing FMDV serotypes A, O, and Asia-1 that are relevant for this region. ELISA titers were assessed at 28, 90, and 180 days after vaccination in cattle of three age groups at the time of initial vaccination: 4–6 months, 6–18 months and ≥ 24 months of age with sheep of all ages.

**Results:**

The 3 age groups of cattle had similarly high levels of immunity with over 90% of the cattle showing a ≥ 50% protective titer 28 days after the first vaccination. By day 90, titers in cattle from the initial 4–18-month age groups dropped below 58% across the 3 serotypes and at or below 80% for the oldest cattle ≥ 24 months. Re-vaccination of cattle at 120 days did improve protective titers but never reached the level of immunity of the first vaccination. Sheep showed a similar rapid drop to less than 50% having a ≥ 50% protective titer at 90 days emphasizing the need for continual revaccination.

**Conclusions:**

The results of this study have important implications for the current FMD vaccine policy in Armenia and improves our understanding of the rapid loss of protective titers over short periods. Since small ruminants are only vaccinated once per year and vaccination titers drop rapidly by 90 days suggests that they are vulnerable to FMD and that vaccination protocols need to be updated. Cattle should continue to be vaccinated every 3–6 months depending on their age to maintain a protective level of antibodies to protect them from FMD. More studies are needed to understand the possible role of small ruminants in the epidemiology of FMD and to evaluate revaccination at shorter intervals. These results show the concerns of rapid loss of protection to both cattle and small ruminants following 1 or more doses of commercial vaccines and that additional vaccines need to be evaluated in both groups to know how often they must be vaccinated to provide full protection. The addition of challenge studies should also be considered to better understand the level of protection as measured by serology and how it relates to protection from challenge. These results should be considered by anyone using these vaccines in cattle and sheep at longer than 3 month intervals.

## Introduction

Foot-and-mouth disease (FMD) is a severe, highly contagious viral disease of livestock that has a significant economic impact [1]. It is a transboundary animal disease that deeply affects the production of livestock and disrupts regional and international trade in animals and animal products. FMD virus (FMDV) is transmitted through direct contact between naïve and infected animals via the exudates of blisters, blood, and saliva or through the contamination of the environment with these infectious fluids [2, 3]. Naive animals can also be infected through ingestion of contaminated feed [4, 5], meat products, and milk [6–8]. Mechanical transfer via fomites such as farm equipment and farm workers and by aerosolization of the virus [9–11]. FMD is caused by an *Aphthovirus* of the family *Picornaviridae* and includes seven serotypes (A, O, C, SAT1, SAT2, SAT3, and Asia-1) which are endemic in many countries worldwide. FMD A, O, and Asia-1 serotypes are endemic in countries bordering Armenia [12, 13].

Monitoring and evaluation of a disease control strategy is a key component of any control strategy and is fundamental to the FMD Progressive Control Pathway (PCP) Plan (Sumption 2012; FAO 2018b). Armenia has been in the Food and Agriculture Organization of the United Nations (FAO) PCP stage 2 (out of 5 stages) since 2008, which is at the stage of implementation of a risk-based control plan. This requires continual monitoring of outbreak strains and evaluating the risks, our level of implementation, and methods of control. To highlight the importance of vaccines for the control of FMD, the FAO and the World Organization for Animal Health (WOAH) published the Post Vaccination Monitoring (PVM) guidelines to advise countries on the principles and suggested procedures for monitoring various aspects of FMD vaccines [14]. This document includes the guidelines for assessments of quality, what constitutes the population-level immunity and coverage, and what are the parameters for the effectiveness of each vaccine. Assessment of vaccine quality is done through small-scale immunogenicity studies [14, 15]. The risk-based assessments are done concurrently with vaccination programs and are implemented through government based national strategy plans.

Governments may dedicate extensive resources to purchasing and administering FMD vaccines, either for routine prophylaxis or in the event of a response to an increase in exposure risk from nearby outbreaks. There are many issues with currently available FMD vaccines including low potency, poor antigenic match between the field and vaccine strains, their relatively short shelf life, reliance on maintenance of the cold chain, a short duration of action, and high population turnover which can limit coverage [16–18]. Despite these constraints, vaccines have been used for successful control of FMD, especially when coupled with additional zoo-sanitary measures [19] including quarantines and restricted movement of animals. The need for FMD vaccines will continue to rise as the global demand increases with the global rise of livestock populations [20, 21].

Armenia is a landlocked country located in the Armenian Highlands of Western Asia. It is a part of the Caucasus region and is bordered by Turkey to the west, Georgia to the north, Azerbaijan to the east, and Iran and the Azerbaijani exclave of Nakhichevan to the south. FMD is not considered endemic in Armenia but is considered at very high risk for incursion of FMD due to its endemicity in neighboring Turkey and Iran. Recently, there have been outbreaks in the Anatolia region of Turkey, which identified the circulation of strain O/ME-SA/PanAsiaQom15 [22]. Additionally, there have been more than 126 FMD outbreaks in large ruminants (LR) and 30 in small ruminants (SR) in Iran in 2022, reporting strains O/PanAsia-2/Ant-10 and A/A05/Far-11 [22]. Thus far in 2022, neither Azerbaijan nor Georgia have reported any cases of FMD [22]. In Armenia, we achieved a > 95% vaccination coverage by the spring vaccination campaign in the buffer zone of RA for LR.

**Fig. 1:**
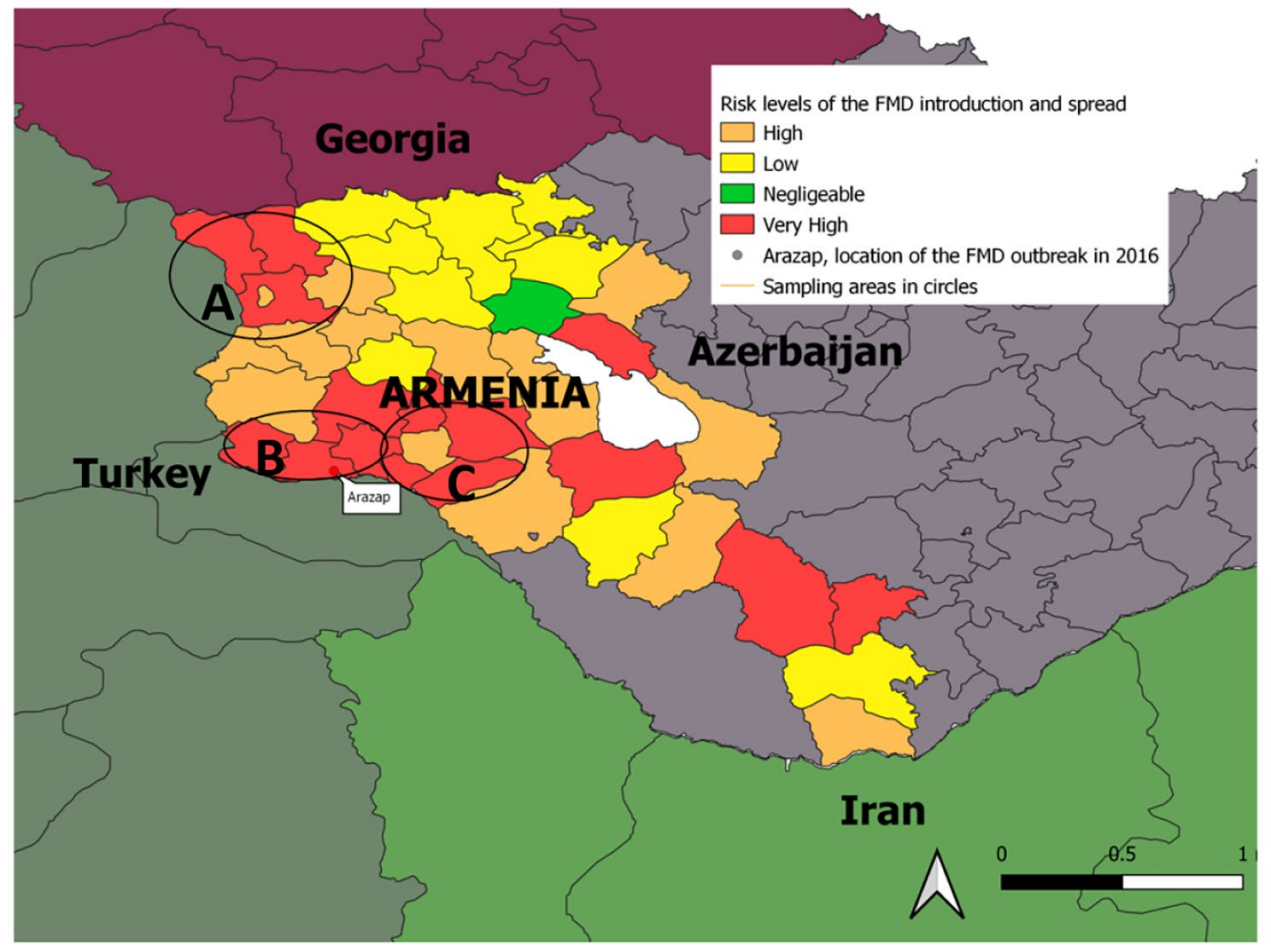
Map of Armenia with surrounding countries showing the Risk levels related to large and small ruminants in relation to road density, distribution of livestock markets, slaughterhouses, and border points in Armenia in 2020. Map was created using QGIS software (Version 3.30.1). Areas within the circles include the sampling areas of Shirak **(A)**, Armavir **(B)**, and Ararat **(C)** Regions. The risk levels are indicated by color and range from very high to negligible. The white flag shows the location of the village of Arazap where the last outbreak of foot and mouth disease (FMD) was identified and resolved in 2016 in Armenia

The last outbreak of FMD in the Republic of Armenia was first reported at the end of 2015 in the Armavir region (Fig. 1). Only one epidemiological unit was involved during the outbreak which included the village of Arazap in the Armavir Region. The village was identified following FMDV disgnosis and extensive prevention and disease eradication measures were implemented. FMD was noted among both cattle and pigs that were previously immunized with a polyvalent vaccine A, O, Asia-1, which included an antigens from the production strains A Iran-2005, O-PanAsia2, and Asia1-Sindh08. Confirmation of FMDV was established following the submission of tissue epithelium to the Scientific Center for Risks Assessment and Food Safety Area (SCRAAFSA, Yerevan, Amenia) which was examined by RT-PCR, ELISA, and CFT, of which the genome and antigen of FMDV type A were detected [23, 24].

In January of 2016, samples were delivered to the WOAH regional reference laboratory for FMD, Federal Center for Animal Health (FGBI “ARRIAH”) (Vladimir, Russian Federation) for a more detailed study. Based on sequencing and phylogenetic analysis, the Armenian isolates were identified as related to the A/G-VII genetic line and are genetically similar to isolates from Turkey and Iran [23].

Additional antigenic studies conducted at the FMD World Reference Laboratory (WRL) (Pirbright, UK), determined that FMD vaccines with antigens (A22IRAQ; A IRAN 2005; A TUR 20/06) cannot protect animals from infection with isolates of the genetic line A/G-VII [25] and a new vaccine was recommended.

Since 2016, following the recommendation of the WRL, WOAH reference laboratory “ARRIAH” and the European Commission for the Control of Foot and Mouth Disease (EuFMD commission) to the Ministry of Health of the Republic of Armenia, we have used a quadrivalent vaccine which also includes the A/Asia/G-VII strain of FMDV. Since then, no clinical confirmed cases have been reported and FMDV surveys are conducted regularly by the state veterinary services for the evaluation of the vaccination program and to identify FMDV circulation.

In order to reach a protective level of immunity in vaccinated animals against FMD, multiple parameters play a role including species composition, susceptible stock density, age of vaccinated animals, vaccine potency, and the type of adjuvant used in the vaccine (oil-adjuvanted vaccines are known to significantly increase humoral immunity and have superior antibody formation). Currently, no specific requirements for achieving a protective level of immunity exist, but at least 80% immune animals in each susceptible population is suggested [14, 26]. The purpose of this study was to assess the FMD immune background of vaccinated animals in three regions of the RA buffer zone, Shirak, Armavir, Ararat, and to provide guidance on optimal vaccine schedules for use in a our national program for FMD vaccination.

## Materials and methods

### Study design

This study was conducted in three parts as part of the national FMD post-vaccination monitoring activities. Protective antibody response and the effectiveness of FMD vaccination studies were based on those prescribed in Sect. 3.2 of the FAO-OIE PVM guidelines [27]. The three studies were: (1) assess the immunity produced by vaccines in cattle of different ages; (2) assess the immunity produced by vaccines in cattle using one and two-dose (revaccination) primary courses; (3) assess the immunity produced by vaccines in sheep. All animals were individually identified to ensure accurate follow-up and were monitored for signs of clinical FMD.

### Vaccine

From 2016 to 2021, Armenia used the tetravalent adsorbed inactivated FMD vaccine which contained strains A-Iran-05; A-G VII; O-PanAsia2, Asia1-Sindh08 and was produced by “ARRIAH”. The initial plan was to provide preventive immunization for 100% of cattle and 50% of sheep in the buffer zone. We administered the vaccine per manufacturers guidelines which includes starting vaccination at 4 months of age in LR and 3 months of age in SR with revaccination every 3 months up to 18 months of age. The dosing directions are 2 ml for cattle and 1 ml for sheep, delivered in the middle part of the neck. The vaccine manufacturer recommends revaccinating immunologically naïve animals (i.e., with no previous exposure, vaccination, or maternally derived antibodies) using either aqueous or oil adjuvant based vaccines.

We calculated the percentage of vaccination coverage by the following calculation: number of vaccinated animals/total number of animals of target population planned to vaccinate*100%.

### Study area

The study was undertaken in the northwest of Armenia, including the Shirak, Armavir and Ararat Regions (Fig. 1), as they are considered high risk areas as they border with Turkey where FMD is endemic [28]. In Armenia, bordering regions and regions with seasonal pastures are also considered as high risk because of the large density of both LR and SR. In the investigated regions, the total animal populations were: 87.396 LR and 58.908 SR (Shirak) 54.074 LR and 113.626 SR (Armavir) and 40.180 LR and 85.180 SR (Ararat).

If a village was selected which did not comply with the required criteria (e.g., sample size per village, species, etc.) the geographically nearest village within the same risk area that complied with the criteria was alternatively chosen by the central level specialists. Animals in the village were selected randomly or, if logistically not possible, by methods that ensure a representative selection, including more than 3 animals per owner.

### Study Population

Blood samples were collected from both LR and SR within the regions of the buffer zone, using the targeted randomized cluster method developed by WOAH to assess the immune status in the population. This allowed us to determine the immune background with an accuracy of 95%. There were 2 groups: risk hotspots (border villages with Turkey, Georgia, Azerbaijan, and Iran) and background (the rest of the country/remaining villages) according to their association with one or more of the risk hotspots.

### Sample size

Within each risk area, the sample size was calculated including both species, as they are considered to share a similar risk. Previous studies have shown that LR and SR are often kept together and analysis of results from a previous serosurvey showed similar non-structural protein (NSP) prevalence [26]. We utilized the online sample size calculation tools http://www.winepi.net and http://www.epitools.ausvet.com to determine the appropriate sample size for the estimated prevalence. The confidence level was set at 95%; with a population size – N of LR + N of SR; and expected prevalence – based on the expected prevalence per risk area: for risk 1–80%; accepted error − 10% agreed.

In the surveyed regions, we identified the zone of highest risk of FMD introduction. For each zone, there were 10 settlements/farms, from which we collected blood samples from 23 LR and 15 SR. This included 10 samples from each from 10 settlements (100 samples) from cattle 0–6 months of age per region (300 for all 3 regions), 7 samples each from 10 settlements (70 samples) from cattle aged 6–18 months per region (210 for all 3 regions), 6 samples each from 10 settlements (60 samples) from cattle older than 24 months per region (180 for all 3 regions), and 15 samples from each of 10 settlements (150 samples) from sheep of any age per region (450 for all 3 regions).

### Sample collection

Sampling was carried out at 28, 90, 180 days after vaccination (Table 1). The sampling began after the autumn vaccination campaign (September-October). The first sampling was in November 2020 with the second in December-January 2021, and the third and last sampling was implemented in March 2021, before starting the spring 2021 vaccination campaign. Sampling was carried out from the same animals and the same locations each time.

**Table 1:** Cattle and sheep sampling timeline for all 3 regions combined at each time point

Age of animals at first vaccination	28 days pv^a^	90 days pv^a^	30 days after revaccination (day 120 pv^a^)	90 days after revaccination (day 180 pv^a^)	180 days pv^a^
4–6 months cattle	300	300	130	130	170
6–18 months cattle	210	210	140	140	70
≥ 24 months cattle	180	180	0	0	180
Sheep	450	450	0	0	450
Total	1140	1140	270	270	870

^a^ post vaccination (pv)

Seromonitoring studies were carried out at the Reference Laboratory for Especially Dangerous Pathogens of the Republican Veterinary and Phytosanitary Laboratory Services Center. The total number of samples collected included 2,070 blood samples from cattle and 1,350 blood samples from sheep from Shirak, Armavir and Ararat regions. On the 90th day after vaccination, blood samples were taken from all of the examined animals and half of the examined 0–18 months old cattle population were revaccinated (Table 1).

All serum samples were taken by venipuncture using either the jugular or caudal (tail) vein depending on species and handling facilities present. Animal blood was collected in serum separator vacutainers with a red cap and delivered to the laboratory in cold boxes within 24 h. Upon arrival in the laboratory, serum tubes were centrifuged, and serum was aliquoted into two individual tubes: 1.5 ml for initial testing and stored at 8 °C and 2ml for long term storage at -20 °C. All data was written on the sample collection forms and the blood tube/vacutainers.

### Field Survey

The “FMD Field Survey Form” was submitted together with the serum to the laboratory. The sample collection forms contained the following epidemiological information: sample number; location; province; district; village; owner name; sex; age in months; month of the last FMD vaccination, vaccine type; remarks: current or previous (when?) presence of FMD clinical signs; distance pasture (by less & more 10 km). All data, including field and laboratory data, were entered into an electronic spreadsheet for analysis. The clinical survey was conducted according to the approved Standard Operative Procedures defined by the SCRAAFSA.

### Diagnostic testing

Serum samples were tested for the presence of antibodies to FMDV serotypes A, O and Asia-1 using the commercially available solid-phase competitive ELISA (SPCE) per the manufacturer’s instructions (Istituto Zooprofilattico Sperimentale della Lombardia e dell’Emilia Romagna (IZSLER), Brescia, Italy) and as recommended in the WOAH Guidelines for Diagnostic Tests and Vaccines for Terrestrial Animals [11].

To evaluate the field effectiveness of vaccination, we considered the ELISA antibody titers of ≥ 1:30 in 80% of animals as sufficient to prevent FMD epizootic. Data was collected in excel and mean log_10_ ELISA titers were calculated for each time point by age groups of cattle and sheep.

## Results

To assess the field effectiveness of FMD vaccination and the immune background of cattle vaccinated against FMD in the FMD buffer zone of Shirak, Armavir and Ararat Regions, blood serum samples were tested by ELISA, 28, 90 and 180 days after vaccination. Animals ˃4 months of age were vaccinated previously as the current Armenian vaccination protocol follows the vaccine manufacturers guidelines for cattle starting at 4 months of age. During the study period, there were no reported outbreaks of FMD in cattle or sheep.

### Immune response in cattle

**Fig. 2:**
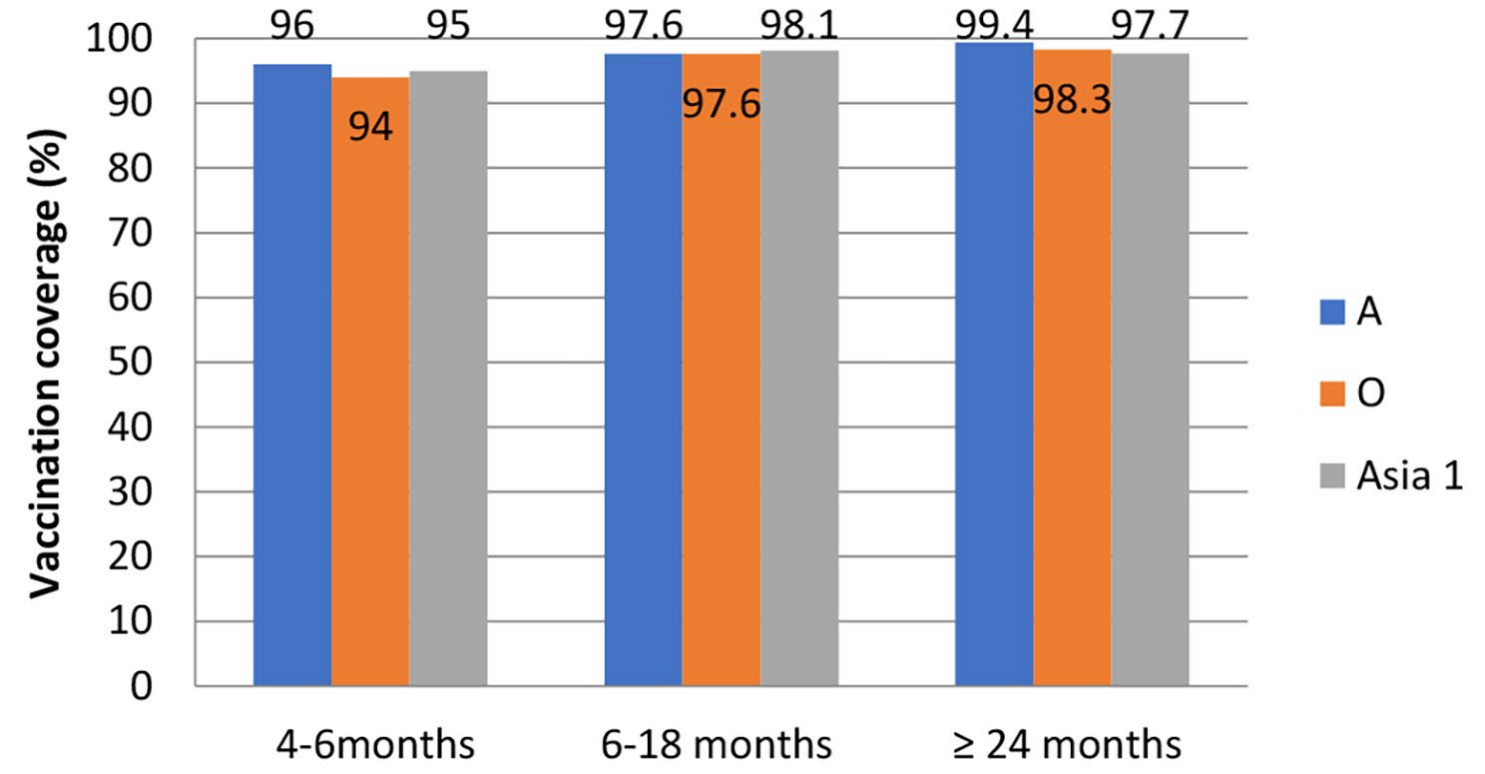
Vaccination coverage (with the protective positive titer of ≥ 50%) for cattle in 3 age groups at time of first vaccination to FMDV serotypes A, O, Asia-1 28 days post vaccination

On the 28th day after vaccination, a high level of immune animals (above 94.0%) to FMDV serotypes A, O, Asia-1 was noted among the cattle population of all age groups (Fig. 2).

**Fig. 3:**
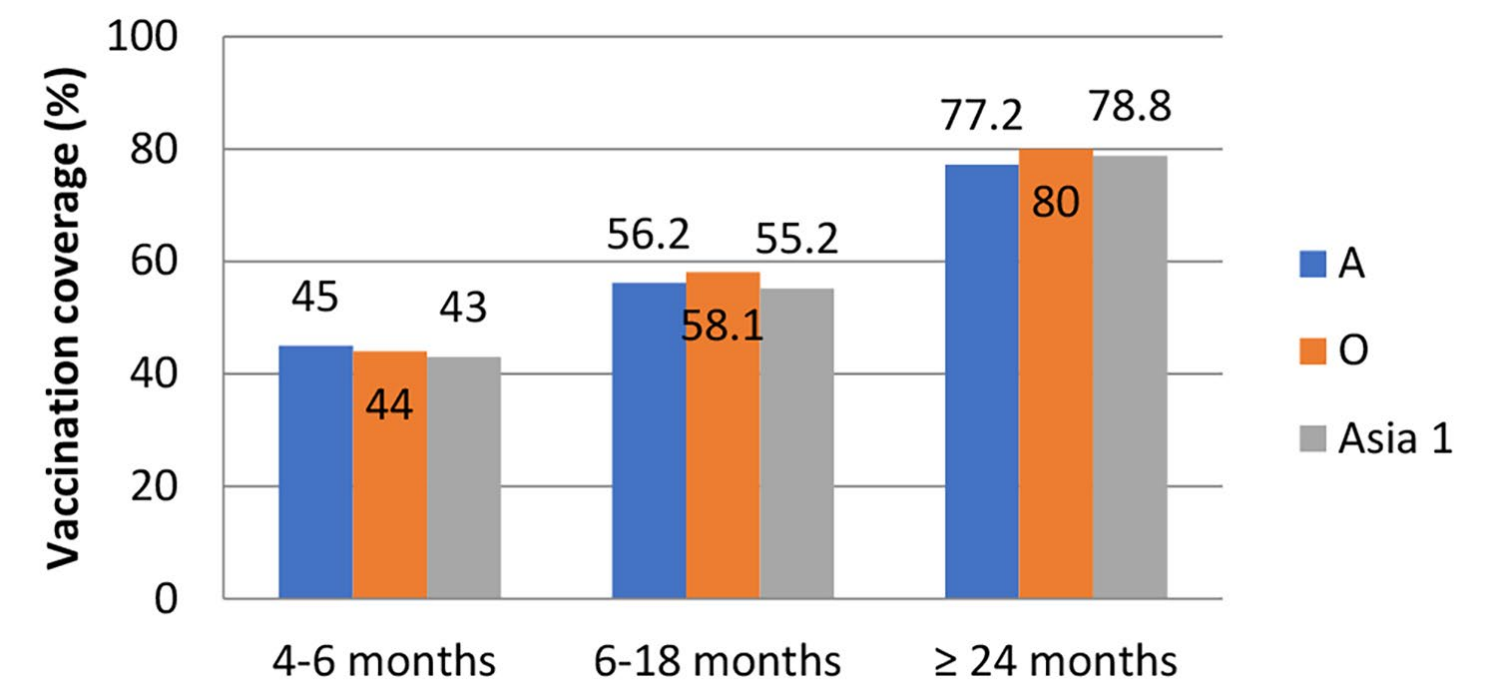
Vaccination coverage (with the protective positive titer of ≥ 50%) for cattle in 3 age groups at time of first vaccination to FMDV serotypes A, O, Asia-1 90 days post vaccination

On the 90th day after vaccination (Fig. 3), the level of immune animals to the three serotypes of FMDV among the examined vaccinated cattle population differed based on the age of the animal. The highest level of immunity (above 77%) was recorded only in animals older than 24 months, and in young animals that received only one vaccination, the level of immunity fell to 43%.

**Fig. 4:**
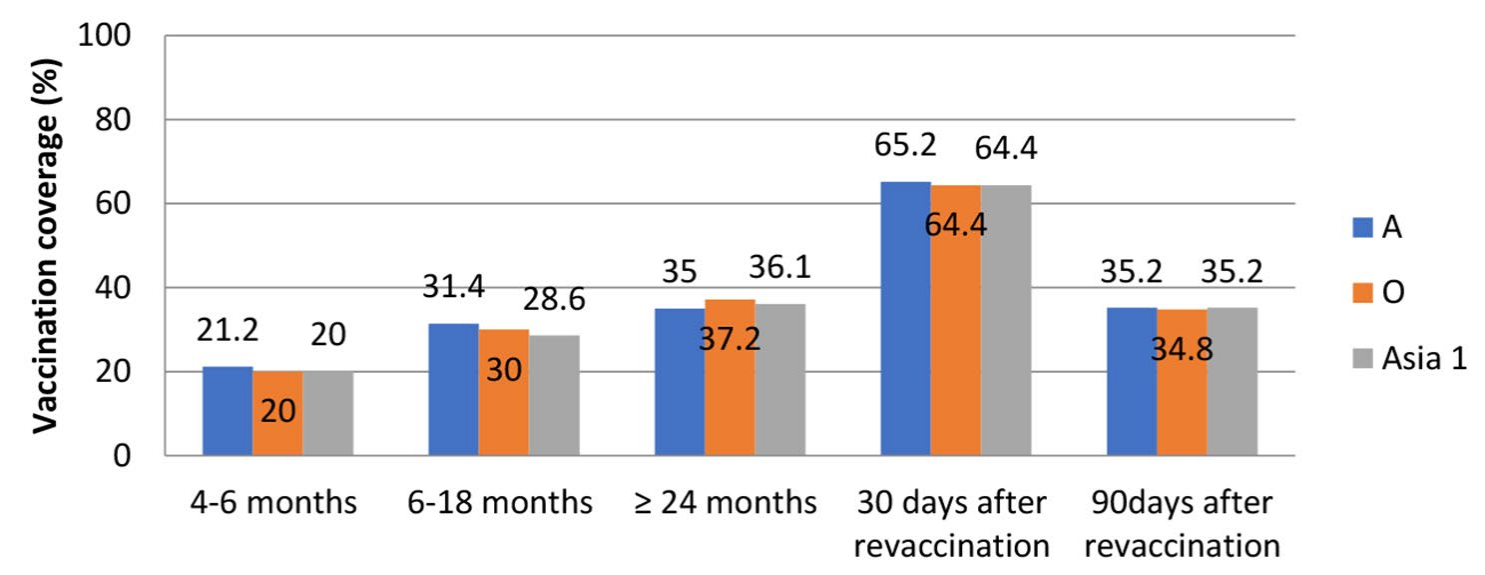
Vaccination coverage (with the protective positive titer of ≥ 50%) for cattle to FMDV serotypes A, O, Asia-1 at 180 days post vaccination in cattle 4–6, 6–18 and ≥ 24 months of age at first vaccination and 30 days (120 days after the first vaccination) and 90 days (180 days after the first vaccination) after revaccination in 4–18 months of age cattle

On the 180th day after vaccination (Fig. 4), the level of immune animals among the cattle population of all age groups fell sharply. As the level of population immunity dropped below 35% after a single vaccination, those cattle that were revaccinated 90 days after the first vaccination showed improvement. For the revaccinated cattle, now between ages of 7–18 months, the level of immunity was significantly higher reaching 63–68%, which indicates that in order to obtain a high level of immunity, repeated vaccinations are required.

### Immune Response in Sheep

**Fig. 5:**
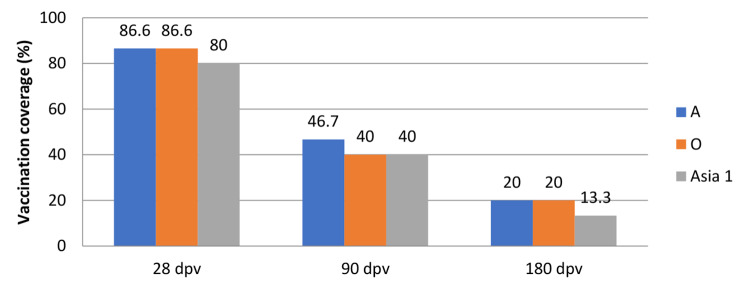
Vaccination coverage (with the protective positive titer of ≥ 50%) of sheep to FMDV serotypes A, O, Asia-1 on days 28, 90, 180 post vaccination (dpv)

A similar picture was observed among the sheep (Fig. 5). The highest level of immune sheep to FMDV serotypes A, O, Asia-1 was observed on the 28th day after vaccination (80-86.6%). On the 90th day, the level of immunity decreased to 40–46%, and on the 180th day, the level of immune animals was 13.3–20%.

**Fig. 6:**
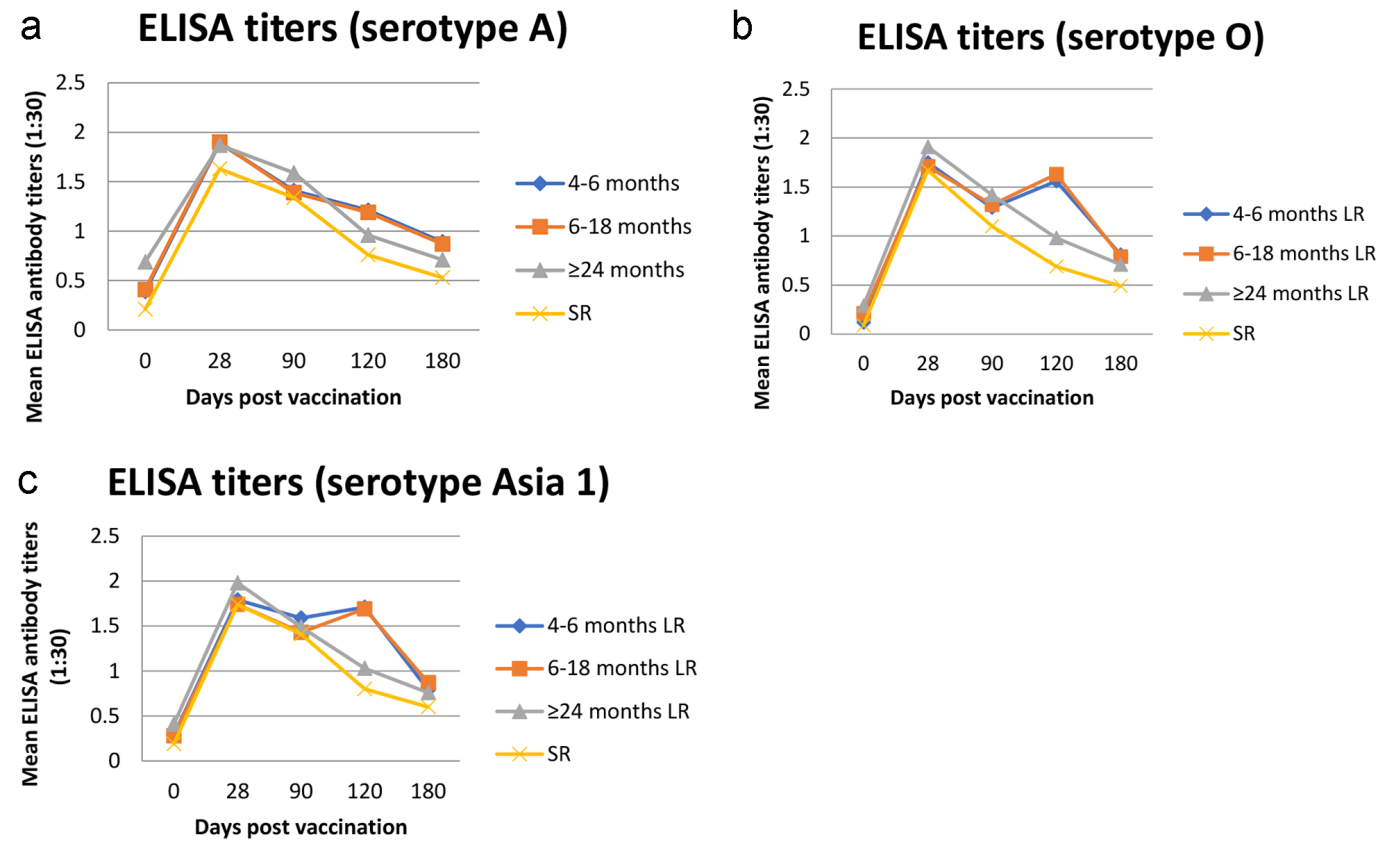
Mean ELISA log_10_ antibody titers for **(A)** FMDV serotype A, **(B)** FMDV serotype O, **(C)** FMDV serotype Asia1 at a titer of 1:30 for cattle (LR) in three age ranges and small ruminants (SR) at all ages 28–180 days post vaccination (s)

For calves vaccinated with the tetravalent adsorbed inactivated FMD vaccine “ARRIAH” and assessed by ELISA against serotype A, O, and Asia 1, all reached a peak mean antibody titers (1:30) at 28 dpv with a range of log_10_ 1.74 to 1.98 across the three age groups (Fig. 6). For calves, by day 90 the ELISA mean titers ranged from log_10_ 1.29 to 1.59. The calves that received the booster vaccination at 90 dpv did show an increase in mean titers from 90 to 120 days for serotypes O and Asia 1 (1.63–1.69 log_10_) that never quite reached the 28 day maximum mean titers still dropped dramatically at day 180 with mean ELISA titers ranging from log_10_ 0.71–0.89 (Fig. 6) For sheep, the mean peak ELISA titers were also at 28 dpv, remained at protective levels to 90 days then dropped even lower than in cattle at 180 days suggesting the need for a booster vaccination.

## Discussion

This study utilized standardized approaches to determine the effectiveness of vaccination and the immune response in calves and sheep following vaccination with the “ARRIAH” FMD vaccine. We sought to determine the protective immunity produced by evaluating ELISA antibody titers at several time points post vaccination and to evaluate if boosting at 3 months improves the immune response and for how long. The goal was to assess the current vaccination policy in Armenia and evaluate if this policy is achieving the best protection from an incursion of FMD into Armenia. The last outbreak of FMD in the Republic of Armenia with the FMD virus A serotype from the A/G-VII genetic lineage was in 2016 and was similar to reported isolates from neighboring Turkey and Iran [23, 25] and is the most recent incursion of a new lineage into Armenia.

**Fig. 7:**
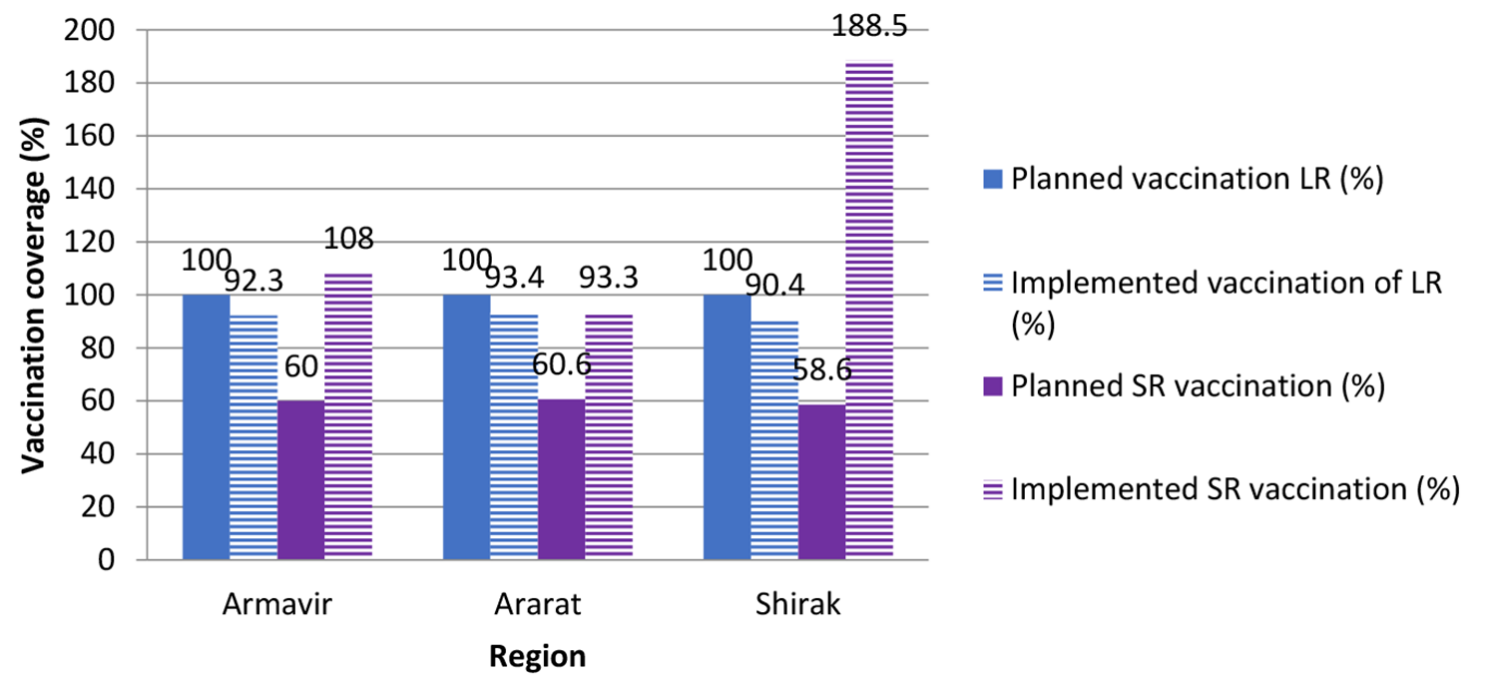
Vaccination coverage of large ruminants (LR) and small ruminants (SR) of Armavir, Ararat and Shirak regions in 2020 based on numbers of animals planned to be vaccinated vs. implemented

Based on the data from the Ministry of Economy and specialists from the SCRAAFSA we have shown that from 2016 to 2020, we implemented > 90% coverage in cattle and > 58% coverage in SR (Fig. 7) for protecting against FMD in Armenia using the “ARRIAH vaccine in the three studied regions. The planned and implemented numbers will vary by year based on the budget provided by the Government of Armenia and the true numbers of animals during implementation.

The ELISA results indicated an overall weaker immune response in calves (4–18 months) after a single vaccination, with a seroconversion rate reaching 90% and log_10_ titers of 1.75–1.89 peaking at 28 dpv followed by a rapid decline of antibodies by day 90. On the 90th day, a decrease in titers of up to 75% of antibodies in cattle ≥ 24 months was found, which largely exceeded the expected protection threshold followed by a rapid decrease, again in younger animals. On the 90th day after vaccination the coverage of protective titer of antibodies in 4–6 months old cattle were 43–45% with titers of log_10_ 1.29–1.59. And after 180 days, antibody titers in all age groups decreased significantly from 19 to 35% with titers of log_10_ 0.71–0.89. Only in revaccinated animals, now aged 7–18 months old, which were re-vaccinated on the 90th day after the first vaccination, showed an increase in the level of immunity (63–68%) 30 days after revaccination with titers of log10 1.19–1.69 which again rapidly decreased to 34–35% by 90 days after revaccination. These results are similar with others using the same “ARRIAH” vaccine in cattle and sheep [29]. They noted a similar decrease in antibody titers at 56 days post single vaccination in various ages of cattle and sheep. We expanded on their work by looking at later time points and evaluating the immune response by ELISA following a booster vaccination. We show, that even following the booster vaccination, the titers still did not reach the initial peak at 28 days and there was no improvement on the sustainment of antibody response at 180 dpv. Others have also shown an early drop in vaccine effective effectiveness with different vaccines but that multiple vaccines could improve the protective coverage in cattle [30].

These results indicate that to maintain the coverage of protection in vaccinated animals, a revaccination course for calves must be included irrespective of the vaccine serotype consistent with previous field-based and experimental observations [17, 31].Others using different FMDV vaccines have shown a longer duration of immunity with higher ELISA titers at 180 days ( [32, 33] including high ELISA values above log_10_ 1.5 at 9 months [32].

Our study does have some limitations when comparing to other studies. We were unable to perform virus neutralization (VN) studies with the corresponding homologous vaccine strains to compare with our ELISA results. We also do not have the capacity to perform challenge studies to confirm that the proposed level of protection of ≥ 80% or log_10_ titers of ≥ 1.5 are truly protective in the event of an outbreak. We also have had no new outbreaks following the implementation of our vaccination campaigns and are unable to account for the true protection afforded in Armenia. Additional studies are also needed in SR as the current vaccination scheme of once yearly is not effective based on our results and it is unknown what role SR play role in the transmission of FMD in Armenia.

What we have shown is that the immunity studies conducted in 2020 confirmed that the polyvalent vaccine “ARRIAH”, does not induce protective and long-term population immunity without revaccination at short intervals. The percentage of immune animals was reduced to 35% under our vaccination plan which increases the risk of incursion of FMD and potential spread of FMD in Armenia. Thus far, our vaccination scheme of cattle has been effective for the prevention of FMD in the buffer zone of the RA as a whole when vaccinating young cattle every 3 months up to 18 months as indicated. Unfortunately, vaccination of older cattle and sheep at longer intervals shows that more regular vaccinations are needed to improve protection of all animals from disease.

Additionally, initial protection is provided to newborn calves who receive anti-FMD virus antibodies in maternal colostrum. The maternally derived antibody (MDA) provides immediate protection against infection with FMDV but can interfere with the development of active immunity following vaccination. However, susceptibility to infection precedes the ability to respond to vaccination in the presence of MDA [34]. Currently available vaccines with aluminum hydroxide adjuvant cannot overcome this inhibitory effect of MDA, and protection of young stock can only be provided by their isolation from exposure to FMDV [35]. Thus, the vaccination of cattle in Armenia should start after 4 months of age [17] with revaccination at approximately 3 month intervals.

Following the outbreak of FMD in Armenia in 2015, and the identification of the circulating strain as A/G-VII prompted Armenia to update their vaccine to include this serotype coverage. Analysis of the FMD epidemic situation in the RA during 2016–2020 indicates that our use of the currently relevant vaccine has improved our control of FMD as there have been no new cases of disease but caution the results by suggesting the increased interval of revaccination. These results have been used to update the FMD control policy in Armenia and show the importance of post-vaccination monitoring and independent assessments of different FMD vaccines to guide vaccination strategies and give confidence among stakeholders in the chosen approach for vaccination. This work adds to our understanding of the lack of long-term immunity against FMD in cattle and should further be evaluated following re-vaccination in SR.

## Data Availability

The datasets analyzed during the current study are available from the corresponding author upon reasonable request.
